# Detection of therapeutic radiation in three-dimensions

**DOI:** 10.3762/bjoc.13.129

**Published:** 2017-07-05

**Authors:** John A Adamovics

**Affiliations:** 1 Department of Chemistry, Biochemistry and Physics, Rider University, 2083 Lawrenceville Road, Lawrenceville, NJ 08648-3099, USA

**Keywords:** dosimeters, leuco dyes, polymers, radiation, triarylmethane synthesis

## Abstract

For over the last twenty years there has been a multitude of sophisticated three-dimensional radiation delivery procedures developed which requires a corresponding verification of the impact on patients. This article reviews the state of the art in the development of chemical detectors used to characterize the three-dimensional shape of therapeutic radiation. These detectors are composed of polyurethane, radical initiator and a leuco dye, which is radiolytically oxidized to a dye absorbing at 630 nm.

## Introduction

Radiotherapy treatment is a complex 3D process, which is the principle treatment modality for most cancers [1]. The two main types of radiation therapy are external beam and internal beam. External beam radiation can be sorted into 2 main types of ionizing radiation: photon (X-rays and gamma rays) and particle radiation (electron, protons, neutrons, and carbon ions) [1]. Internal radiation therapy can be delivered by either a solid radioactive source (brachytherapy), or a liquid radiation source placed near or inside the cancerous area.

In the last decade the sophistication and complexity of radiation therapy treatment has increased dramatically. Advances have been so swift that an imbalance has arisen with verification technologies (dosimeters) with sufficient capability to verify complex treatments and ensure accurate, safe implementation [2]. There have been reports of high failure rates for complex radiation treatments [3–4]. These concerns and others have led many to recognize an urgent need to strengthen the foundations of quality assurance (QA) in radiation therapy [3–4]. One of the most frequently used dosimetric tools is two-dimensional radiochromic film where a color is formed upon reaction with ionizing radiation [5].

**Scheme 1:**
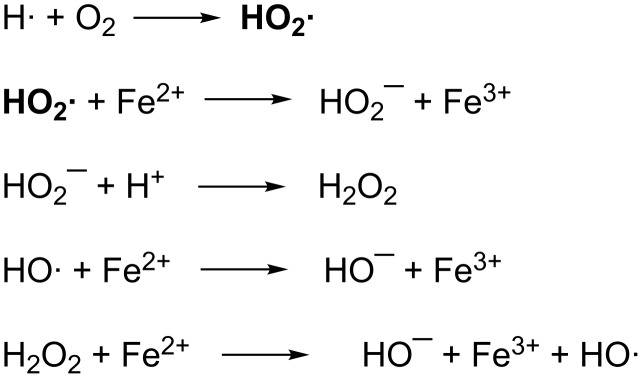
Ionizing radiation reactions in the Fricke dosimeter.

A ferrous sulfate solution (Fricke solution) where ferrous (Fe^2+^) ions are oxidized to ferric ions (Fe^3+^) was the first chemical approach to quantifying ionizing radiation [6]. During irradiation water is decomposed to reactive HO· and H· radicals which further react with oxygen to produce the hydroperoxy radical which oxidizes the ferrous ions (Scheme 1) [7–8]. The ferric ion generates a blue color that is quantified spectrophotometrically.

**Figure 1:**
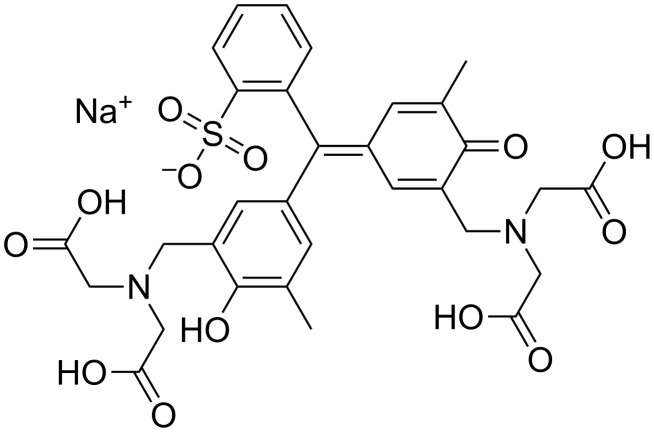
Structure of xylenol orange.

In order to stabilize the geometric dose information in the Fricke solution aqueous based gel matrices containing the chelator xylenol orange were reported [9–11] with the molecular structure shown in Figure 1. When analyzed spectrophotometrically, a non-irradiated ferrous/agarose/xylenol orange (FAX) gel shows visible-light absorption at 440 nm; after exposure to ionizing radiation, there is an increase in absorption at 585 nm. Even though diffusion has been diminished it continues to be an issue [12].

These diffusion limitations were overcome in a gel matrix by the polymerization of acrylamide with *N*,*N*’-methylenebisacrylamide and various monomers to yield a cloud like precipitate in the aqueous gel [13]. Due to the nature of their radical chemistry, polymer gel dosimeters have several limitations. They are susceptible to atmospheric oxygen inhibiting the polymerization processes. Irradiated dosimeters scatter light during optical scanning. The solutions are toxic, require 24 hours to equilibrate, and require a container to maintain the dosimeter shape [13].

Interest in a 3D dosimeter made of a transparent plastic was initially reported in 1961 [14]. The ideal dosimeter would be firm in structure and tissue equivalent [14]. This review describes such a 3D dosimeter, which we have been studying since 2004, composed primarily of the polymer polyurethane containing a radiochromic leuco dye and a radical initiator [15].

## Review

### Leuco dyes and radical initiators

Our initial studies focused on a broad class of compounds referred to as leuco dyes which switch between two chemical forms of which one is colorless. The transformations are caused by the in put of energy either from heat, light or change in pH [16]. The leuco dyes by themselves are not oxidized at clinical radiation doses. Consequently, radical initiators were necessary to promote the transformation. A variety of leuco dyes and radical initiators were screened for response to ionizing radiation. Initially the most promising leuco dye was leucomalachite green (LMG) which is a *N*,*N-*dimethyl-substituted triarylmethane (DTM) [17].

**Scheme 2:**
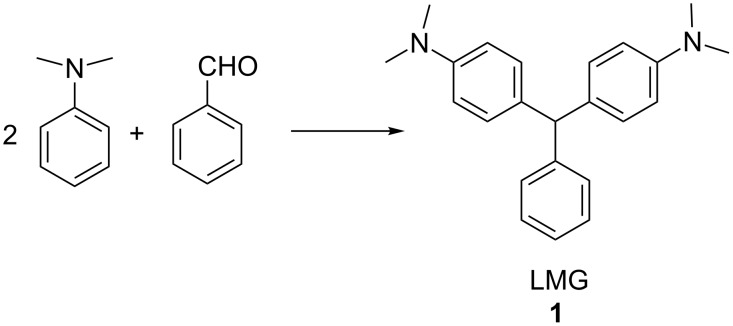
Sulfuric acid/urea promoted synthesis of LMG.

Triarylmethanes (TAMs) have wide ranging commercial, technological and medical applications [17]. In mechanistic chemistry, a triarylmethane demonstrated the first observable organic radical species [18]. TAMs were first synthesized using the Baeyer condensation in 1877 where one equivalent of aryl aldehyde is reacted with 2 equivalents of an electron-rich aromatic compound such as *N*,*N*-dimethylaniline [19] (Scheme 2). This reaction is usually carried out in the presence of various acids [16,20–35]. Microwave radiation procedures have also been reported [36–37].

We prepared several DTMs (Table 1) and measured their respective sensitivities to radiation [38–40] and confirmed structures by ^1^H and ^13^C NMR [20–36]. Progress of the reaction to form the DTMs was conveniently achieved by monitoring the ^1^H NMR spectra, in which the representative CHO proton singlet of the starting aryl aldehyde (ca. 11 ppm) diminishes as the characteristic singlet of the methine DTM product (ca. 5.5 ppm) grows during the course of the reaction. The conformational structure of a DTM has been experimentally determined by computational modeling and vibrational spectra to be twisted much like a three-bladed propeller [20]. We found that numerous other aromatic aldehydes gave good results while highly hindered aryl aldehydes, such as pentamethylbenzaldehyde, 2-fluorenecarboxaldehyde, 9-anthracenecarboxaldehyde, and 1-pyrenecarboxaldehyde, yielded no detectable DTM products. *N*,*N*,*N*-trialkyl-substituted triarylmethanes (e.g., leuco crystal violet) were also synthesized using the above synthetic procedures (e.g., 4-dimethylaminobenzaldehyde as starting aryl aldehyde) but these were too easily oxidized during fabrication of the dosimeters to be useful. Other *N*,*N*-dialkylaniline derviatives, (diethyl, dipropyl and dibutyl) provided the corresponding DTMs. However, only the *N*,*N*-diethyl derivatives proved to be useful as leuco dyes in our dosimeters.

**Table 1:** Synthesized DTBs and their LMG (**1**) relative radiation dose sensitivity.

DTB	Relative dose sensitivity	DTB	Relative dose sensitivity

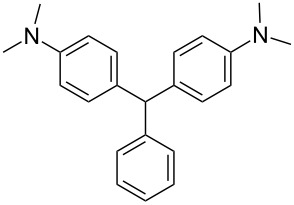 **1**	100	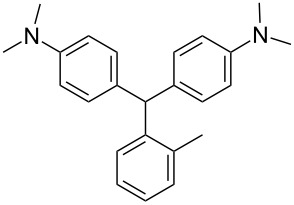 **5**	400
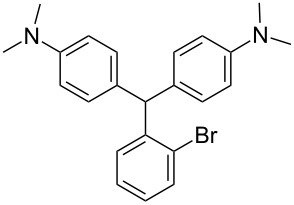 **2**	450	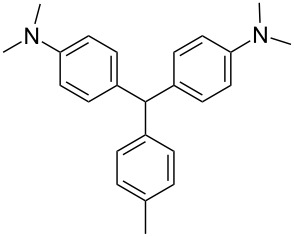 **6**	200
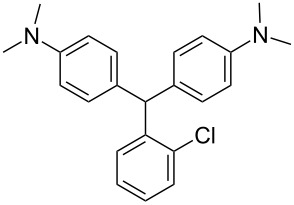 **3**	340	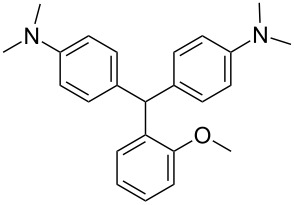 **7**	200
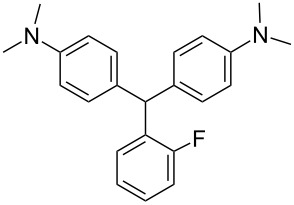 **4**	60	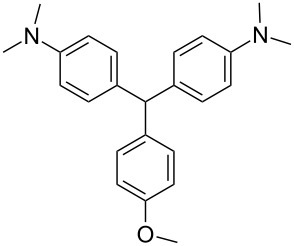 **8**	350

### Radical initiators

In order for the dosimeter to be reactive to a clinical radiation dose a radical initiator is required. The most effective class of initiators are halocarbons while azo- and peroxide-based initiators were unstable to the temperatures generated during the manufacture of the dosimeters [17,41]. The dose sensitivity was found to be consistent with the bond energy of the carbon–halogen bond. The observed sensitivity was in the order R_3_C–I > R_3_C–Br > R_3_C–Cl [42–44]. Due to the high electron density of radical initiators containing iodine even at relatively low concentrations (100 mM) result in dosimeters that are not tissue equivalent [43–45].

#### Polyurethane

Acrylic, epoxy, polycarbonate, polyester, polystyrene, polyurethane, polyvinyl chloride and silicone were the common transparent plastics that were evaluated as potential 3D dosimeter matrices [17]. Polyvinyl chlorides and silicones were not further considered since their effective atomic number is not tissue equivalent. Acrylates, polyesters, polystyrenes and polycarbonates were also eliminated due to the relatively high exotherms created (>100 °C) during polymerization which prematurely oxidize the leuco dyes and rendered the dosimeter product unusable due to high background color. Epoxy resins, which use basic curatives, oxidize leuco dyes making them inappropriate for use as dosimetric matrices. This left the polyurethanes as the most viable option.

**Figure 2:**
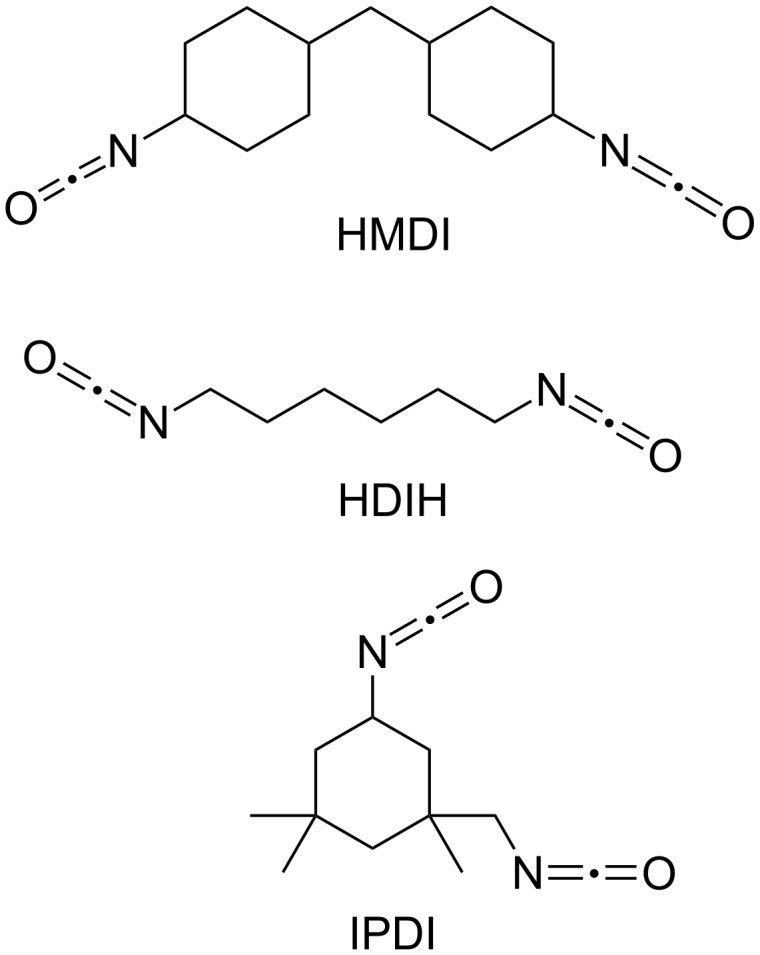
Aliphatic diisocyantes HMDI, HDI, IPDI.

Transparent polyurethane starting materials are commercially available in two parts where part A is typically a mixture of dicyclohexylmethane-4,4'-diisocyanate (HMDI, Figure 2) and it’s polyether prepolymer (CAS 531-70-03-9). While part B is a polyether or polyester polyol mixture which is proprietary [46]. Other aliphatic diisocyanate also used are 1,6-hexamethylene diisocyanate (HDI) and isophorone diisocyanate (IPDI) [47]. The polymerization reaction is exothermic and the rate of curing is dependent on the temperature, concentration of reactive groups, total volume of the reactants and type and concentration of metal catalyst. A number of metals have been studied in the polymer reaction but the most frequently used are dibutyltin dilaurate and phenyl mercuric acetate [48–49]. Besides catalyzing the polyurethane reaction metals (such as Bi, Sn, and Zn) at 1–3 mM have also have demonstrated an effect on the dose sensitivity of the dosimeter [50].

The formulation procedure involves solubilizing the reactants, introducing the resulting solution into a mold; then allowing the polymer to cure at ambient temperature (>20 °C) in a pressure tank (30–60 psi). Performance of the reaction under pressure eliminates formation bubbles of carbon dioxide which is formed as a byproduct of the reaction of adventitious moisture with the diisocyanate. The degree of hardness of the dosimeter can be contolled by the type of polyol and catalyst utilized. Hardness ranging from rigid to tissue-like can be achieved [46]. The urethane reaction also tolerates up to relatively high addition (50%) of various solvents such as butyl acetate and most phthalates.

### Dosimeter radiolysis

**Figure 3:**
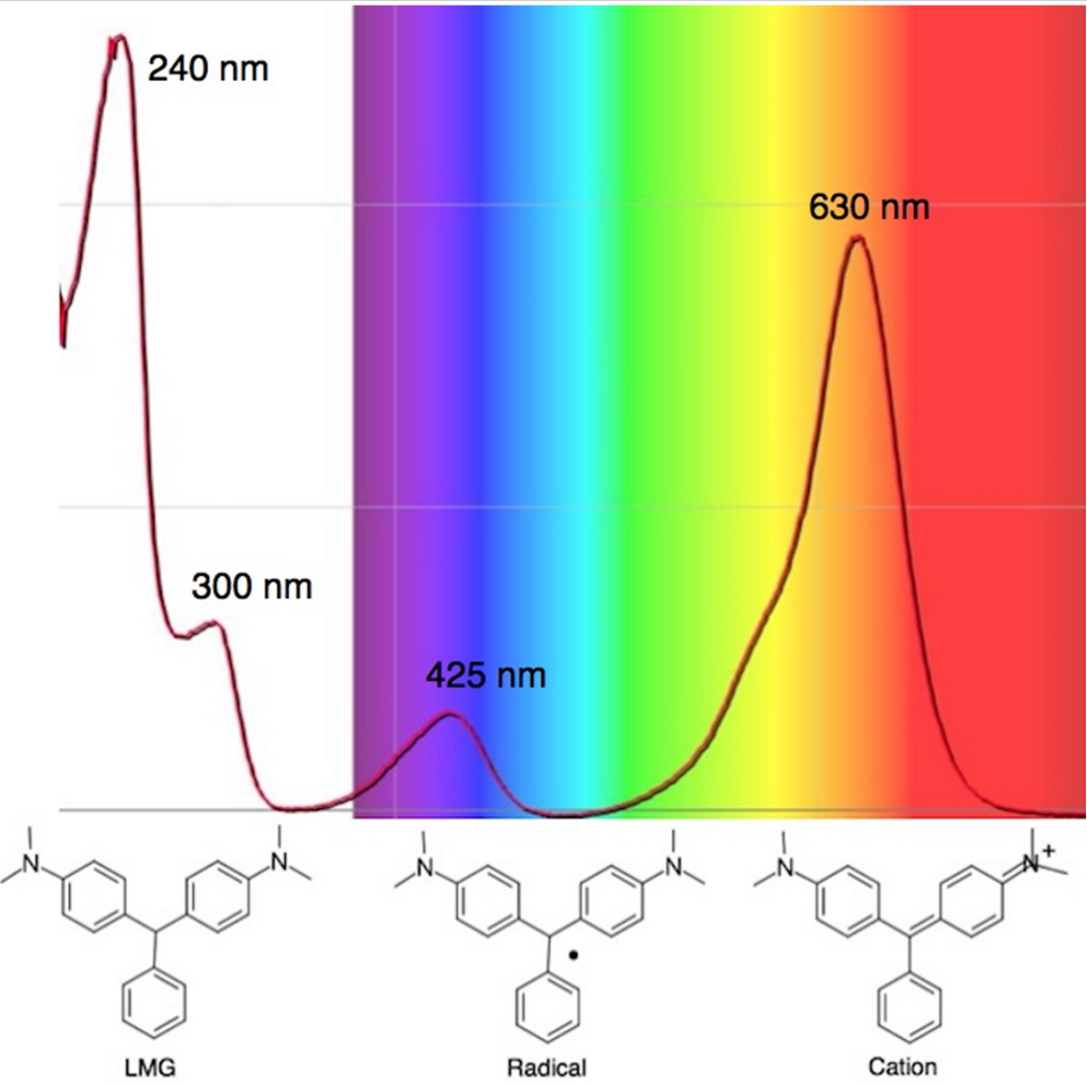
Absorption spectrum of irradiated leucomalachite green.

The initial radiolytic reaction is the dissociation of the radical initiator and subsequent reaction with LMG to create a radical which absorbs at ca 425 nm followed by the formation of the malachite green cation absorbing at 630 nm [51–52] (Figure 3).

The density of the radical is primarily on the central carbon with some charge distribution to the nitrogen substituents [51–53]. Radical stability is largely due to steric protection [53] of the central carbon which is consistent with what is observed for the radiation dose sensitivities of the eight DTMs which varied from 4.5 times greater than LMG for the most sterically hindered bromide derivative **2** to the least for the *ortho*-fluoride **4** with 0.6 less dose sensitivity than LMG (Table 1). This is also consistent for the *ortho*-methyl derivative **5** being more dose sensitive than it’s *para*-methyl derivative **6**. There are electronic contributions of the *para*-methyl **6** in stabilizing the radical relative to **1** which has no *para-*substituent. For the *ortho*- and *para*-methoxy derivatives, **7** and **8**, respectively, the interpretation of the steric and electronic contributions is not as straight forward since **8** is more dose sensitive than **7** and almost that of **5**. The addition of polar aprotic solvents such as DMSO also enhances the dose sensitivity [52].

The other important characteristic is the post-irradiation color stability where in general those DTMs with the greatest steric hindrance near the methine carbon provide the greatest color stability. In contrast the *para*-substituent DTBs have demonstrated the most facile color fading [39]. A combination of singlet oxygen and light is thought to be the cause of bleaching of DTBs [54] even though for these dosimeters the effect is minimal [55].

### Dosimeters

**Figure 4:**
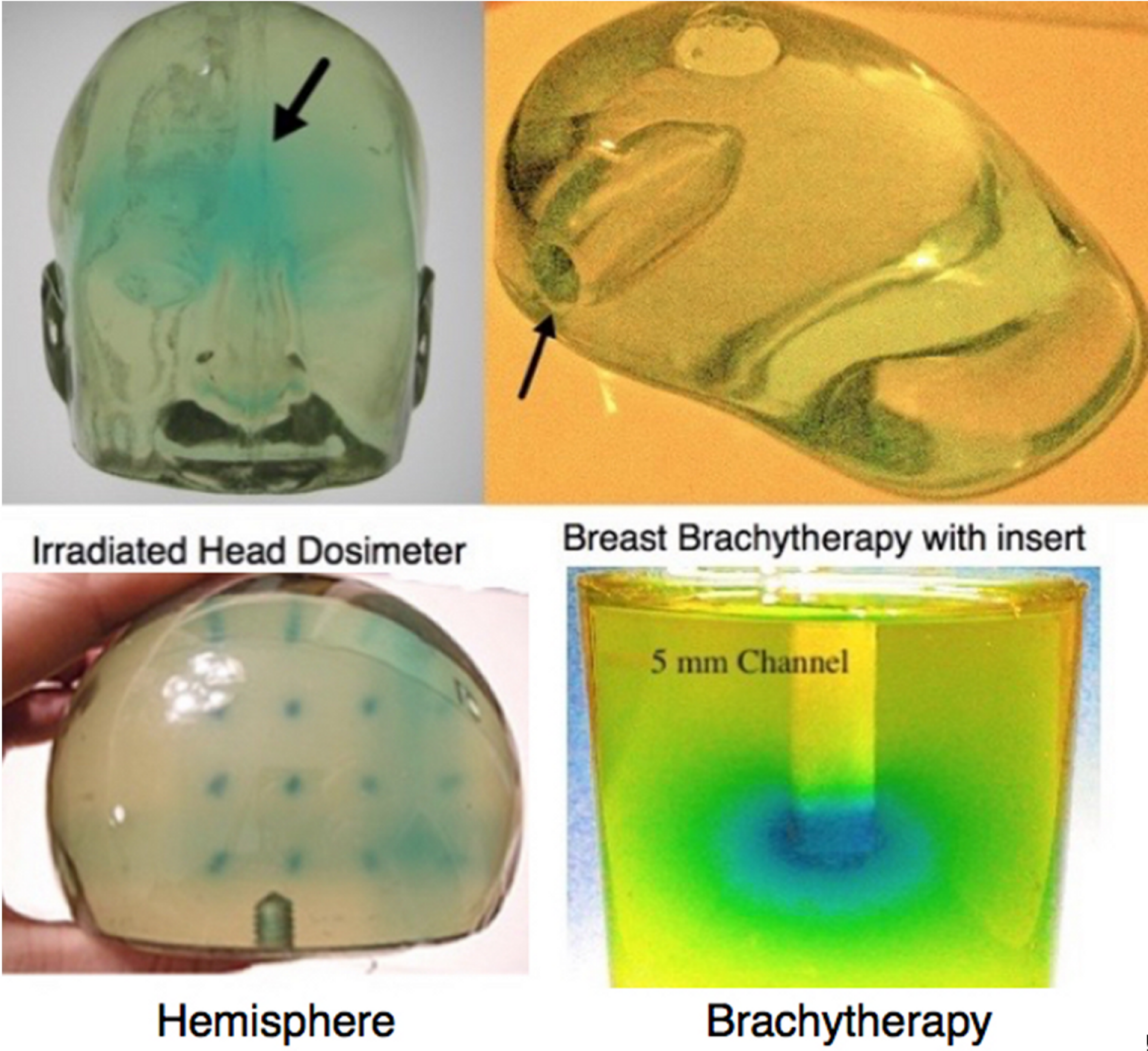
3D dosimeters fabricated in our lab for a variety of radiation therapies. Top left a head dosimeter (12 kg); on the right a breast dosimeter with an inset for brachytherapy; bottom left an irradiated hemisphere; bottom right a cylindrical brachytherapy dosimeter with 5 mm channel for insert the radiation seed.

Due to the versatile nature of the dosimeter system described above virtually any shaped dosimeter can be fabricated as illustrated below (Figure 4).

### Optical computed tomography (OCT) scanning

**Figure 5:**
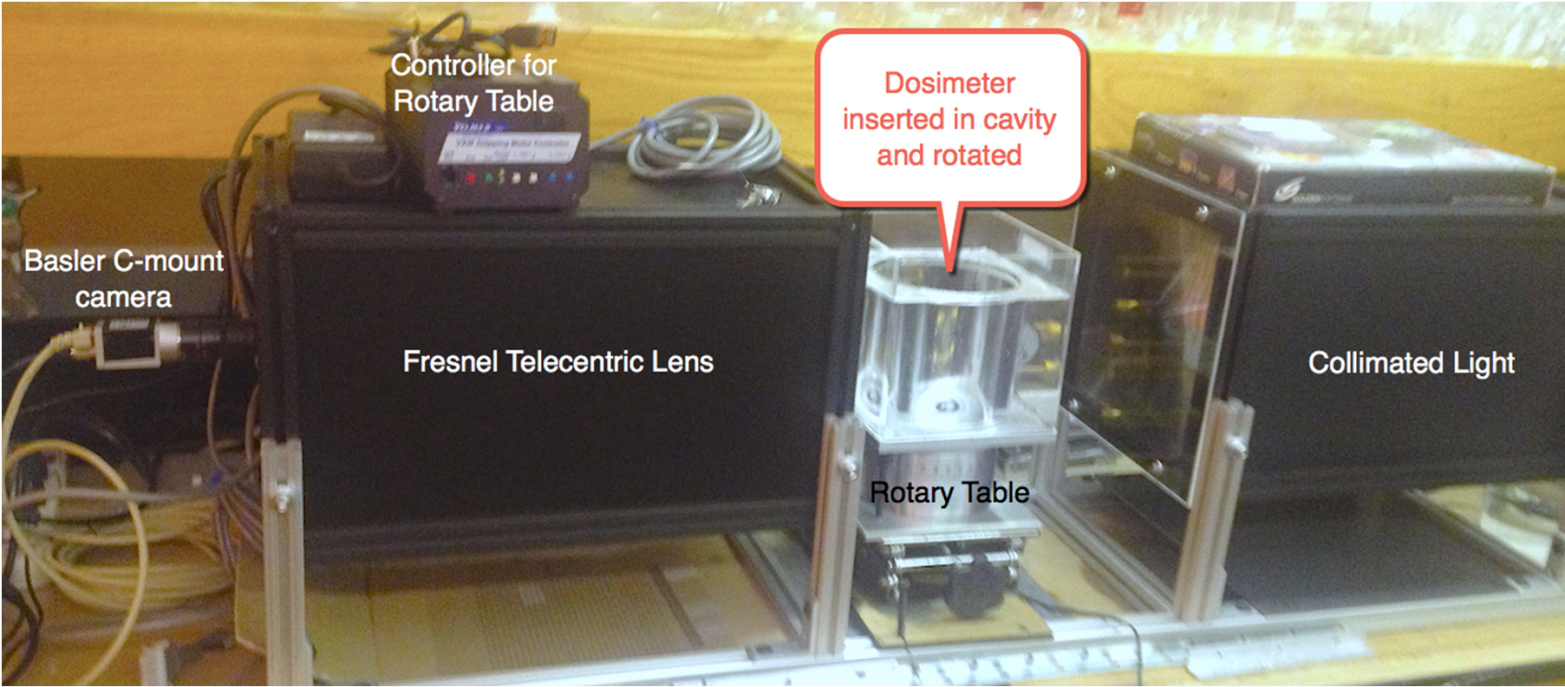
OCT scanner used in our lab to create 3D images.

In order to create a 3D image of the irradiated dosimeter, it is placed inside a tank of refractive index matching solvent and on one side of the tank there is a collimated light source that shines through the dosimeter, a stepper motor rotates the dosimeter 360 degrees as the C-mount camera /lens [56] captures images at 1 degree increments (Figure 5). The 360 2D images are reassembled to give a full 3D image of the color density within the dosimeter [56].

### Overview

Due to the DTMs that differ in their physiochemical properties and polyurethanes that are commercially available a wide array of clinical related radiation treatment applications have been demonstrated. These include internally delivered radiation in which a cavity is created in the dosimeter for placement of radioactive seeds, deformable dosimetry in which the elastic properties of the dosimeter are manipulated to mimic those of human tissue, and reusable dosimetry [39,43–44]. Clinical research dosimeter adaptions have also made possible the study of alternative treatment approaches such as the addition of nanoparticles containing metals to the dosimeter to evaluate enhanced radiation effects [57] and utilizing mice in evaluating radiation treatment plans [58].

## Conclusion

Over the last twelve years there has been significant progress made in developing chemical-based three-dimensional radiation detection systems but as of this review these dosimeters are primarily used in clinical research settings. This is partially due to the lack of a viable commercially available OCT scanner and availability of alternative semi-3D radiation measuring systems that interpolate 3D radiation dose distributions based on a sparse array of point detectors [59] which does not measure true 3D.

## References

[1] (2017). National Cancer Institute, Radiation Therapy for Cancer.

[2] Molineu A, Hernandez N, Nguyen T, Ibbott G, Followill D (2013). Med Phys.

[3] Starkschall G (2010). J Appl Clin Med Phys.

[R4] 4Herman, M., *Medical Radiation: an Overview of the Issues.* On behalf of the American Association of Physicists in Medicine (AAPM), 2011.

[5] Soares C G (2006). Radiat Meas.

[6] Fricke H, Morse S (1927). Am J Roentgenol, Radium Ther Nucl Med.

[7] Fricke F S, Hart E J, Attix F H, Roesch W C (1966). Chemical Dosimetry. Radiation Dosimetry.

[8] Schreiner L J (2004). J Phys: Conf Ser.

[9] Gore J C, Yang Y S (1984). Phys Med Biol.

[10] Bero M A, Gilboy W B, Glover P M, Keddie J L (1999). Nucl Instrum Methods Phys Res, Sect A.

[11] Baldock C, Harris P J, Piercy A R, Healy B (2001). Australas Phys Eng Sci Med.

[12] Maryanski M J, Gore J C, Kennan R P, Schulz R J (1993). Magn Reson Imaging.

[13] Doran S J (2009). Appl Radiat Isot.

[14] Potsaid M S, Irie G (1961). Radiology (Oak Brook, IL, U S).

[15] Adamovics J, Maryanski M J (2006). Radiat Prot Dosim.

[16] Muthyala R (1997). Chemistry and applications of leuco dyes.

[17] Adamovics J, Jordan K, Dietrick J (2006). J Phys: Conf Ser.

[18] Gomberg M (1900). J Am Chem Soc.

[19] Fischer O (1877). Ber Dtsch Chem Ges.

[20] Bardajee G R (2011). Beilstein J Org Chem.

[21] Halimehjani A Z, Shamiri E V, Hooshmand S E (2016). J Appl Chem Res.

[22] Muthyala R, Katritzky A R, Lan X (1994). Dyes Pigm.

[23] Ritchie C D, Sager W F, Lewis E S (1962). J Am Chem Soc.

[24] Alvaro M, Garcia H, Sanjuán A, Esplá M (1998). Appl Catal, A.

[25] Chalk A J, Halpern J, Harkness A C (1959). J Am Chem Soc.

[26] Zhang Z-H, Yang F, Li T-S, Fu C-G (1997). Synth Commun.

[27] An L-T, Ding F-Q, Zou J-P (2008). Dyes Pigm.

[28] Bardajee G R, Jafarpour F (2009). Cent Eur J Chem.

[29] Jafarpour F, Bardajee G R, Pirelahi H, Oroojpour V, Dehnamaki H, Rahmdel S (2009). Chin J Chem.

[30] Rao H S P, Rao A V B (2016). Beilstein J Org Chem.

[31] Nambo M, Crudden C M (2015). ACS Catal.

[32] Nair V, Thomas S, Mathew S C, Abhilash K G (2006). Tetrahedron.

[33] Li Z, Duan Z, Kang J, Wang H, Yu L, Wu Y (2008). Tetrahedron.

[34] Guzmán-Lucero D, Guzmán J, Likhatchev D, Martinez-Palou R (2005). Tetrahedron Lett.

[35] Malpert J H, Grinevich O, Strehmel B, Jarikov V, Mejiritski A, Neckers D C (2001). Tetrahedron.

[36] Khosropour A R, Esmaeilpoor K, Moradie A (2006). J Iran Chem Soc.

[37] Reddy C S, Nagaraj A, Srinivas A, Reddy G P (2009). Indian J Chem, Sect B.

[38] Alqathami M, Adamovics J, Benning R, Qiao G, Geso M, Blencowe A (2013). Radiat Phys Chem.

[39] Juang T (2015). Clinical and Research Applications of 3D Dosimetry.

[40] Adamovics J (2007). Three-dimensional shaped solid dosimeter and method of use. U.S. Pat. Appl..

[41] Miyaji T, Tokita S, Tachikawa T, Azuma C (2001). J Photopolym Sci Technol.

[42] Denisov E T, Denisova T G, Pokidova T S (2005). Handbook of free radical initiators.

[43] Alqathami M, Blencowe A, Qiao G, Butler D, Geso M (2012). Radiat Phys Chem.

[44] Alqathami M (2013). Novel 3D radiochromic dosimeters for advanced radiotherapy techniques.

[45] Singh V P, Badiger N M (2014). J Med Phys.

[46] (2017). BJB Enterprises.

[47] Delebecq E, Pascault J-P, Boutevin B, Ganachaud F (2013). Chem Rev.

[48] Saunders K J (1988). Polyurethanes. Organic Polymer Chemistry.

[49] de Lima V, da Silva Pelissoli N, Dullius J, Ligabue R, Einloft S (2010). J Appl Polym Sci.

[50] Alqathami M, Blencowe A, Qiao G, Adamovics J, Geso M (2012). Radiat Phys Chem.

[51] Ayyangar N R, Tilak B D, Venkataraman K (1971). Basic Dyes. The Chemistry of Synthetic Dyes.

[52] Bobrowski K, Dzierzkowska G, Grodkowski J, Stuglik Z, Zagorski Z P, McLaughlin W L (1985). J Phys Chem.

[53] Hicks R G (2007). Org Biomol Chem.

[54] Oda H (2005). Dyes Pigm.

[55] Alqathami M, Blencowe A, Ibbott G (2016). Phys Med Biol.

[56] Thomas A, Newton J, Adamovics J, Oldham M (2011). Med Phys.

[57] Alqathami M, Blencowe A, Yeo U J, Doran S J, Qiao G, Geso M (2012). Int J Radiat Oncol, Biol, Phys.

[58] Bache S T, Juang T, Belley M D, Koontz B F, Adamovics J, Yishizumi T T, Kirsch D G, Oldham M (2015). Med Phys.

[59] Feygelman V, Zhang G, Stevens C, Nelms B E (2011). J Appl Clin Med Phys.

